# Microstructure and development of the dermal ossicles of *Antarctopelta oliveroi* (Dinosauria, Ankylosauria): A complex morphogenetic system deciphered through three‐dimensional X‐ray microtomography

**DOI:** 10.1111/joa.14159

**Published:** 2024-11-05

**Authors:** Sophie Sanchez, Armand de Ricqlès, Jasper Ponstein, Paul Tafforeau, Louise Zylberberg

**Affiliations:** ^1^ Department of Organismal Biology, Subdepartment of Evolution and Development Uppsala University Uppsala Sweden; ^2^ European Synchrotron Radiation Facility Grenoble France; ^3^ CR2P, UMR 7207CNRS/MNHN/Sorbonne Université, Muséum National d'Histoire Naturelle, Bâtiment de Géologie Case Postale 48 Paris France; ^4^ Humboldt‐Universität Zu Berlin Berlin Germany; ^5^ Museum für Naturkunde Berlin Berlin Germany

**Keywords:** histology, 3D virtual histology, ankylosaur, dermal ossicles, metaplasia, neoplasia, SEM

## Abstract

Ankylosaurs were a group of heavily armored non‐avian dinosaurs (Dinosauria, Ankylosauria), represented by a relatively abundant fossil record from the Cretaceous of North and South America. Their dermal skeleton was characterized by large osteoderms whose development and functional role have been largely investigated. However, interstitial small ossicles, forming between these osteoderms, have been far more overlooked and it remains unknown whether they were formed through the ossification of a preexisting fibrous matrix of connective tissue (i.e., metaplasia) or by a cell‐induced differentiation of new fiber bundles followed by mineralization (i.e., neoplasia sensu (Zeitschrift für Wissenschaftliche Zoologie, 1858, 9, 147)). Here, we propose a hypothesis on the developmental origin of these small ossicles in the ankylosaurian *Antarctopelta oliveroi* using light microcopy, scanning electron microscopy and three‐dimensional virtual histology through propagation phase‐contrast synchrotron radiation micro‐computed tomography (PPC‐SRμCT). Ossicles are located in the dermis. They are composed of two layers: (1) a thin external layer, and (2) a thick basal plate, composed of collagen fiber bundles, which forms the main part of the ossicle. The external layer is made of a smooth, vitreous mineralized tissue that does not look like bone. The basal plate, however, is of osseous origin. In this basal plate, the collagen fiber bundles are organized in two orthogonal systems: one horizontal—observable in cross‐sections—and one vertical—observable in the primary plane of sections sensu (Journal of Vertebrate Paleontology, 2004, 24, 874). The horizontal system is itself composed of successive layers of collagen fiber bundles arranged into an orthogonal plywood‐like structure. The bundles of the vertical system radiate from the center of the ossicle at the level of the transition between the external layer and the basal plate and run towards the periphery of the basal plate. Their thickness increases from the center of the ossicle towards its periphery. Numerous bundles of the vertical system form thin threads that interweave and penetrate within the thick bundles of the horizontal system. Our new data suggest that the ossicles were at least partially formed by metaplasia, that is, through the ossification of a preexisting fibrous matrix of connective tissue. This process was probably supplemented by a cell‐induced differentiation of new fiber bundles laid down prior to their incorporation into the fibrous system and its mineralization. This process looks more akin to neoplasia sensu (Zeitschrift für Wissenschaftliche Zoologie, 1858, 9, 147) than to metaplasia. Consequently, metaplastic and neoplastic processes may coexist in these ossicles with a possible differential expression during ontogeny.

## Graphical abstract

Series of images of the dermal ossicle of *Antarctopelta oliveroi* (Dinosauria, Ankylosauria) illustrating (A) the 3D distribution of the vascular mesh in the ossicle; (B) the large volume of the numerous cells in periphery; (C) the radial orientation of the fiber bundles in the main core of the osteoderm (called basal plate); (D) the orthogonal organization of the crystallite matrix. These different patterns suggest that both metaplastic and neoplastic processes coexisted to form this ossicle.

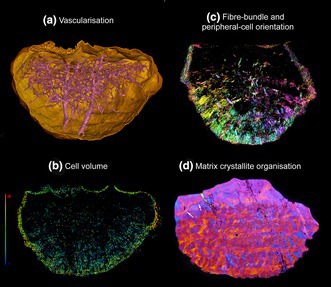

## 1 INTRODUCTION

The present study is the continuation of preliminary histological observations carried out by de Ricqlès et al. (2001) on the small ossicles found in the dermal armor remains of the Antarctic ankylosaurian dinosaur (Gasparini et al., 1987, 1996; Salgado & Coria, 1996) identified as *Antarctopelta oliveroi* (Salgado & Gasparini, 2006). The bone histology of *Antarctopelta* has been reviewed recently (Cerda et al., 2019) via a complete survey of available elements of the endo‐ and dermal skeletons, including large osteoderms and small ossicles. De Ricqlès et al. (2001) evidenced a complex three‐dimensional fibrillar organization of the ossicles and questioned the hypotheses of their formation. Osteoderms and ossicles can be formed through two distinct processes: (1) metaplasia; and (2) neoplasia sensu Müller (1858). The question was whether the ossicles are formed on a template of preexisting fibrous tissue (i.e., metaplastic bone, Haines & Mohuiddin, 1968; Francillon‐Vieillot et al., 1990) or directly—that is, without a preexisting template (as for neoplastic bone sensu Müller, 1858)—or based on both processes (metaplasia and neoplasia), either simultaneously or perhaps at different developmental times.

Ossicles are dermal skeletal elements characterized by a delayed onset of development, compared with other parts of the dermal skeleton such as the cranial skeleton, gastralia, the turtle carapace, and plastron (e.g., Vickaryous & Hall, 2008; Vickaryous & Sire, 2009). The development of ossicles starts in juvenile individuals, rather than in embryos, from a differentiated tissue instead of an embryonic mesenchyme (Chen et al., 2014; Vickaryous et al., 2015; Vickaryous & Hall, 2008). Ossicles, like other dermal elements, develop at the interface between the outermost loose dermis, the *stratum superficiale*, and the deeper more densely packed dermis, the *stratum compactum*, where collagen fiber bundles are organized in successive layers (Francillon‐Vieillot et al., 1990). In ossicles, the organization of the mineralized fibrillar fabric closely resembles that of the dermal connective tissue suggesting a metaplastic process (Beresford, 1981). Concerning osteoderm development, metaplastic ossification (Haines & Mohuiddin, 1968) consists in the mineralization of the dermis and its direct transformation into hard tissue without a mesenchyme primordium of osteoid and osteoblasts. This was thought to be the prominent mode of formation of osteoderms in extant and extinct amniotes (e.g., Hill, 2005; Levrat‐Calviac & Zylberberg, 1986; Reid, 1996; Scheyer et al., 2007; Vickaryous & Hall, 2008; Williams et al., 2022; Zylberberg & Castanet, 1985).

Authors who have published recently on the structure and development of ankylosaurian osteoderms (including ossicles) agree with the idea that metaplastic ossification of the dermis is the main mechanism of formation of those elements (e.g., Bellardini & Cerda, 2017; Cerda et al., 2018; Cerda, Desojo, et al., 2015; Cerda & Powell, 2010; Ponce et al., 2017; Scheyer et al., 2014; Scheyer & Sander, 2004), in spite of some contradictory evidence (de Ricqlès et al., 2001).

The purpose of the present contribution is to identify the microstructural peculiarities of the ossicles reassessed in light of new histological data obtained thanks to recently developed three‐dimensional techniques. This will help to determine their origin and to get a better understanding of the osteogenic mechanisms linked to their development in this ankylosaurian dinosaur.

## 2 MATERIALS AND METHODS

### 2.1 Material

**FIGURE 1:**
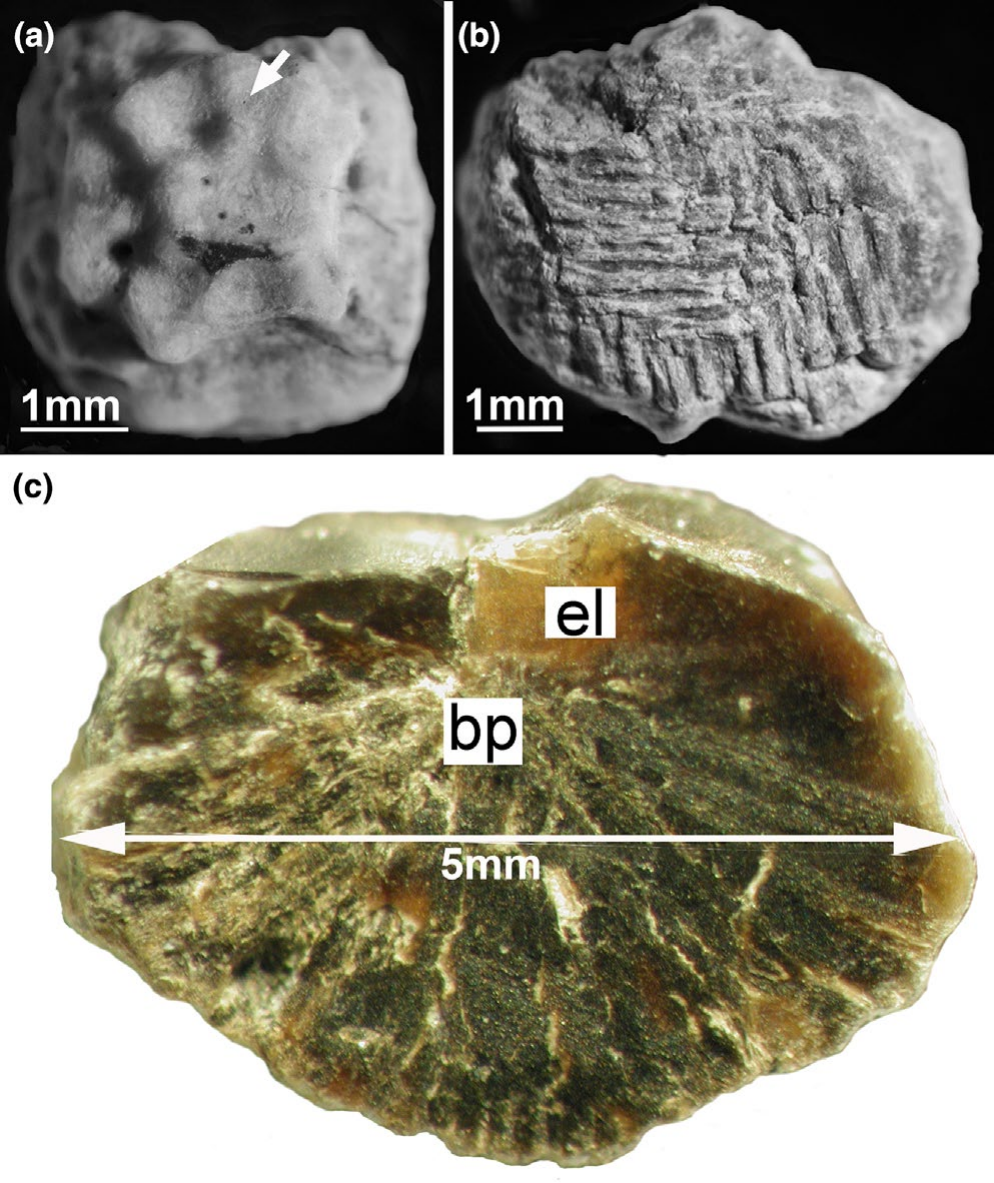
Isolated ossicle (Museo de La Plata MLP 86‐X‐28‐1). (a) External surface. The arrow points to the opening of a vascular canal. (b) Basal surface showing parallel ridges and furrows organized in an ordered system. (c) Vertical fracture of an ossicle. The external layer is composed of a translucent vitreous material that obviously differs from the thick and dense basal plate. bp, basal plate; el, external layer.

The present study was carried out on six small ossicles scattered in the rock matrix and forming a mosaic between larger elements of the semi‐disarticulated dermal skeleton of the holotype *A. oliveroi* (Museo de La Plata MLP 86‐X‐28‐1, Argentina) from the Late Cretaceous of the Antarctic Peninsula (Gasparini et al., 1987, 1996), that were recently described histologically (Cerda et al., 2019). Ossicles were sectioned in the rock matrix for histological examination. Some isolated ossicles freed from the matrix were available. Their orientation could be determined based on the fact that the superficial surface, even if ornamented, is smoother (Figure 1a) than the region supposedly embedded in the skin and crenulated by the fiber pattern (Figure 1b). They were prepared for histological, SEM, and 3D investigations using propagation phase‐contrast synchrotron radiation micro‐computed tomography (PPC‐SRμCT) at the European Synchrotron Radiation Facility (ESRF, France).

### 2.2 Methods for paleohistological examination

Paleohistology is the study of ancient tissues. This research field emerged during the 19th century (Agassiz, 1833–1843; Owen, 1840–1845) and has since provided strong bases for understanding the emergence, evolution and development of fossil hard tissues (e.g., de Buffrénil, de Ricqlès, et al., 2021; de Ricqlès, 2011; de Ricqlès et al., 2004; Padian & Lamm, 2013) including scales, osteoderms and ossicles (e.g., Cerda, Desojo, et al., 2015; Clarac et al., 2020; Meunier & Brito, 2004; Scheyer & Sander, 2004; Schultze, 2016; Vickaryous & Sire, 2009; Witzmann, 2011; Zylberberg et al., 2010). Here, we use two traditional techniques for understanding the paleohistology of the ankylosaur ossicle: light microscopy and scanning electron microscopy.

#### 2.2.1 Light microscopy

Three ankylosaurian fossil ossicles were prepared according to the standard methods of ground sections for light microscope observations outlined by Chinsamy and Raath (1992). Slabs were embedded in stratyl resin and sectioned using a sawing machine (Isomet) for histological examination. The ground sections were polished down to a thickness of about 80 μm and observed using two separate set‐ups: transmitted light (brightfield) microscopy and cross‐polarized light (XPL) with a Nikon Eclipse E66 POL and with a Zeiss Axiovert 35 equipped with Nomarski Differential Interference Contrast (DIC). When needed, a gypsum filter was used to reveal the microstructures in colors.

Ossicles were vertically sectioned displaying the superficial/external layer at the top and the basal plate below. This vertical plane corresponds to the primary plane of section proposed by Scheyer and Sander (2004). Horizontal sections were also made orthogonally to this primary plane of section.

For histological examination of thin sections of isolated ossicles, three ossicles were cleaned with acetone, embedded in Epon under vacuum conditions in plastic molds. These blocks were sectioned using a sawing machine (Isomet) and oriented to obtain thin vertical ground sections that are free slices of about 15 μm thick. A drop of Epon was poured on each side of the cut surfaces and allowed to polymerize at 60°C. After polymerization, the resin forms a thin transparent layer on the surface sections. The sections were examined and photomicrographed using transmitted and polarized light and DIC.

#### 2.2.2 Scanning electron microscopy (SEM)

Two well‐preserved ossicles, isolated from the rock matrix, were vertically broken. They were steeped in a 10% citric acid solution at room temperature to obtain cleaned surfaces. The samples were then washed in distilled water, dehydrated in 100% ethanol, air‐dried, glued on a metal support, and coated with evaporated gold. They were observed in a Jeol‐SEM‐35 scanning electron microscope at 25 kV.

#### 2.2.3 Three‐dimensional virtual histology

Virtual histology is an emerging technique based on propagation phase‐contrast synchrotron radiation micro‐computed tomography (PPC‐SRμCT) for imaging fossils without damaging the specimens (e.g., Giles et al., 2013; Haridy et al., 2021; Sanchez et al., 2012, 2021; Tafforeau et al., 2006, 2007; Tafforeau & Smith, 2008). It has the additional advantage of providing information in three dimensions (3D) to complement our understanding of vertebrate hard‐tissue evolution (e.g., Brazeau et al., 2020; Chen et al., 2016; Davesne et al., 2021; Estefa et al., 2020, 2021; Giles et al., 2013; Jerve et al., 2016; Kamska et al., 2018; Mondéjar‐Fernández et al., 2014; Qu et al., 2017; Rücklin & Donoghue, 2015; Sanchez et al., 2014, 2016; Smith et al., 2010). Volume, orientation, and density can be measured from tomographic data (Davesne et al., 2020; Sanchez et al., 2010, 2013).

This technique was used on one of the ankylosaur ossicles (Museo de La Plata MLP 86‐X‐28‐1). It was imaged at the beamline ID19 of the European Synchrotron Radiation Facility (ESRF, France) following the protocol developed for virtual bone histology (Sanchez et al., 2012). The fossil was scanned with an energy of 20.5 keV in monochromatic conditions using a single bounce multilayer monochromator (a single crystal 2.5 nm period W/B_4_C multilayer monochromator). The beam was pre‐filtered with 3 mm of aluminum to limit the heat load on the crystal and cut the total reflection on the polished substrate at low energy. A decoheror was used to blur the beam structures while keeping enough coherence and a high‐quality beam. The gap of the undulator U32u was set at 11.60 mm. A FReLoN (fast readout low noise) HD2k CCD detector (Labiche et al., 2007) associated to a microscope (magnification 10X) with a 6.2 μm‐thick GGG:Eu (gadolinium gallium garnet doped with europium) scintillator was used to image the sample at a voxel size of 1.4 μm to visualize the collagen fiber bundles, cell lacunae, and vascular canals. 5000 projections were taken in half‐acquisition over 360 degrees, that is, with a shift from the center of rotation over 360 degrees to increase the lateral field of view. The time of exposure was of 0.1 s. The sample was set to a propagation distance of 100 mm to increase the phase contrast. The data were reconstructed using a single distance phase retrieval approach based on a modified version of the algorithm of Paganin et al. (2002) (Sanchez et al., 2012).

The 3D segmentation of the osteoderm was made using VGStudio MAX version 3.0 software (Volume Graphics Inc.). The 3D fiber orientation, cell volume, and cell distribution in the ankylosaurian ossicle were investigated for understanding the developmental origin of the ossicle. The orientation of the fiber bundles and the cell volume measurements were made using two modules of VGStudio MAX: respectively, “fiber composite material analysis” and “porosity/inclusion analysis.”

#### 2.2.4 Terminology

“Osteoderm” is a generic term that most often refers to dermal mineralized elements larger than a few millimeters. They present notable variations in shape, size, and ornamentation; their morphologies, distinctive among genera, and species, are diagnostic for ankylosaurs (Arbour & Currie, 2014; Brown, 1908; Carpenter, 2001; Cerda et al., 2019; Coombs Jr, 1990; Scheyer & Sander, 2004; Vickaryous et al., 2015).

“Ossicles,” whose size is less than a few millimeters (Blows, 2001; Ford, 2000), are globular, compact structures (Barrett et al., 2002; Cerda et al., 2019; Cerda & Powell, 2010; de Ricqlès et al., 2001; Paulina‐Carabajal et al., 2021). They apparently do not exhibit the taxon‐specific characters hypothesized for the larger osteoderms, at least among the derived nodosaurids and ankylosaurids (Burns & Currie, 2014; Burns & Vavreck, 2014). Ossicles form a mosaic of elements infilling the interstitial spaces between actual osteoderms (Arbour et al., 2013, 2014; Barrett et al., 2002; Carpenter, 2004; Cerda & Desojo, 2010; Cerda & Powell, 2010; D'Emic et al., 2009; Molnar, 2001) thereby permitting supple movements of the body and limbs (Carpenter, 1997; de Ricqlès et al., 2001).

The terms “dorsal” and “ventral” refer to the anatomical directions of the body (Burns & Currie, 2014). Regarding the morphological terminology, Scheyer and Sander (2004) described the large ankylosaur osteoderms as composed of “external and internal cortices,” that is, with dense bony layers. Because the nature of the external layer in our ankylosaur ossicle remains unclear (i.e., probably not of osseous nature), we have decided to use the generic term “external layer*”* instead of “external cortex” (Scheyer & Sander, 2004) for the outermost/superficial part of the osteoderm facing the body surface. We will use the term “basal plate” instead of “internal cortex” (Scheyer & Sander, 2004) for the deep part embedded in the skin and oriented inwards as the shape and developmental pattern of this region of the ossicle does not suggest an evenly deposited cortex. The terminology of bone histology described by Francillon‐Vieillot et al. (1990) and de Buffrénil, de Ricqlès, et al. (2021) is used throughout the text.

The term “structural fibers*”* refers to the closely packed bundles of mineralized collagen fibers which compose dermal bones such as osteoderms (e.g., Bellardini & Cerda, 2017; Burns & Currie, 2014; Cerda & Powell, 2010; Hayashi et al., 2010; Ponce et al., 2017; Scheyer & Sander, 2004; Witzmann, 2009; Witzmann & Soler‐Gijón, 2010).

## 3 RESULTS

### 3.1 Morphological observations

The ossicles were found on the external surface of a dorsal rib, and in one case, associated with larger and flat osteoderms (Salgado & Gasparini, 2006). They are relatively small compared to those of most ankylosaurs (e.g., *Minmi*, Molnar, 2001; Salgado & Gasparini, 2006). Their shape is approximately square to polygonal (de Ricqlès et al., 2001). In spite of their small size (each side ranging from 2 to 6 mm), the orientation of the ossicles could be identified based on the obvious differentiation between opposite surfaces observed on isolated samples (as mentioned in the Material section). We identified the smoother surface as the external surface of the ossicle (Figure 1a) whereas the opposite surface, that is convex, is considered as its deep surface (Figure 1b). The external surface, even though smoother, bears tubercles and grooves forming a lumpy superficial surface (Figure 1a). Holes observed on this surface correspond to the opening of channels containing blood vessels (Figure 1a).

**FIGURE 2:**
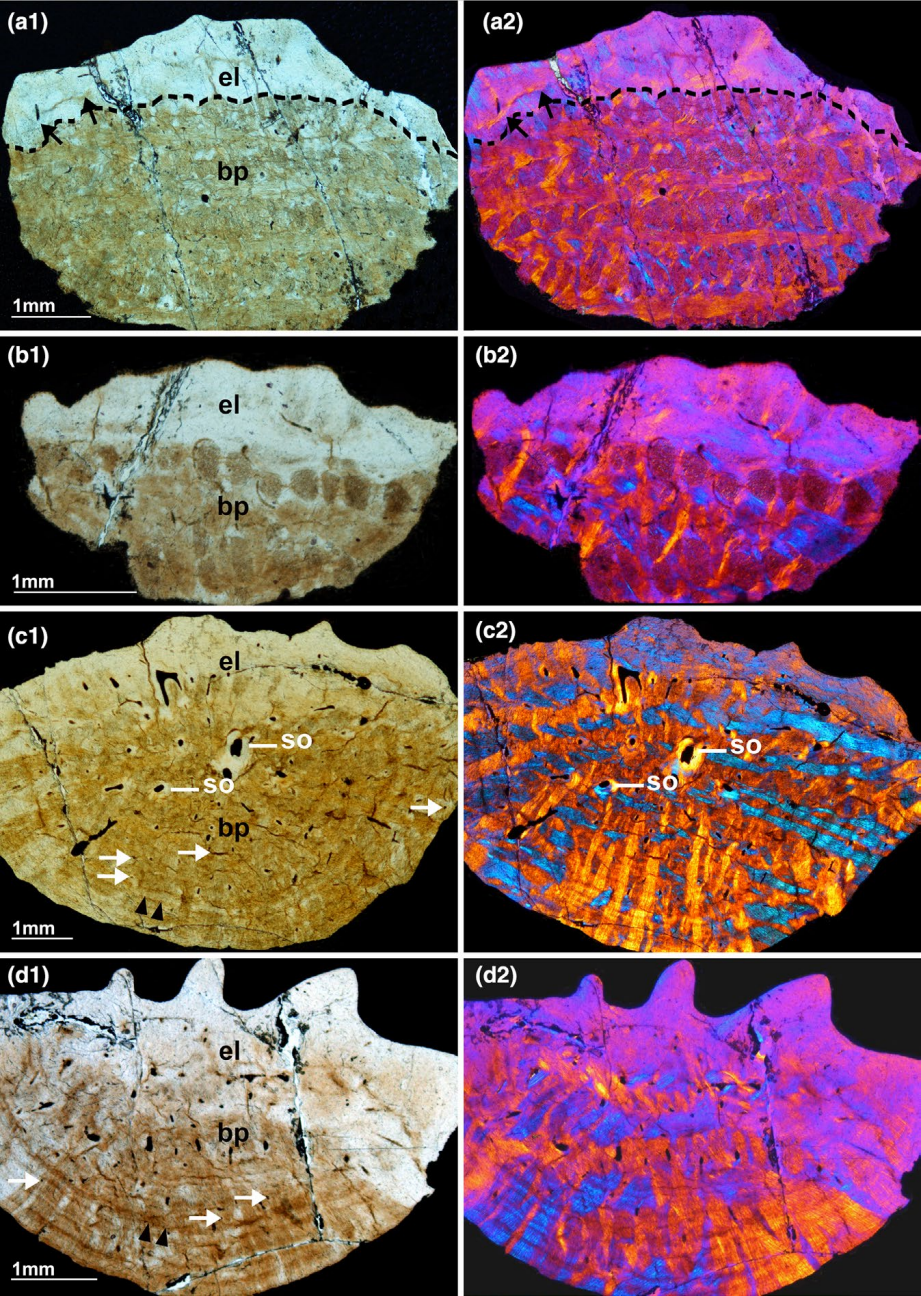
Vertical sections crossing the external layer and the basal plate of ossicles (Museo de La Plata MLP 86‐X‐28‐1). (a_1_–d_1_) Transmitted light (brightfield) microscopy. (a_2_–d_2_) Cross‐polarized light (XPL) microscopy with a gypsum filter. The dashed line in (a_1_,a_2_) separates the external layer from the basal plate. Note the differences in the fiber bundle organization depending on the orientation of the sections. The sections (a,b) perpendicular to the outer and inner surfaces of the ossicles show two sets of structural fibers running perpendicularly whereas oblique sections (c,d) show a complex network of interwoven fiber bundles. The vascularization of the ossicle is represented by thin vascular canals (white arrows) surrounding larger central canals and secondary osteons (c_1_,_2_). In sections (a_1_,_2_), the black arrows point the unconformity resulting from the local action of erosion processes between the external layer and the basal plate. In sections (c_1_,d_1_), Cyclical Growth Marks (CGMs, double arrowheads) are parallel to the basal surface of the ossicles. bp, basal plate; el, external layer; so, secondary osteons.

The ornamentation of the external surface, resulting from sinuous ridges and valleys, does not seem to be the result of superficial processes of erosion/remodeling, as seen among crocodilians (de Buffrénil et al., 2015) but from differential deposition rates (Figure 1a) (similarly to the differential mineralization front at the origin of the dermal bone ornamentation of the temnospondyls *Mastodonsaurus* and *Metoposaurus*, Witzmann, 2009). The basal surface shows parallel ridges and furrows organized in an ordered system (Figure 1b). A fracture in the vertical plane of an ossicle shows a clear‐cut structural change between two consecutive parts. The external layer appears as a translucent, vitreous smooth surface (el in Figure 1c). By contrast, the basal plate is composed of a compact heterogeneous material and shows a rough surface (bp in Figure 1c). On thin sections, the external layer and the basal plate can be well differentiated based on their matrix pattern and color (dashed line in Figure 2a_1_,a_2_). Nevertheless, the relationships between the external layer and the basal plate, in some vertical sections, reveal actions of erosion processes, with a general unconformity between the external layer and the basal plate at the periphery of the ossicles (Figure 2a_1_,a_2_).

### 3.2 Histological observations

#### 3.2.1 General structural organization of the ossicle

Vertical sections, observed under transmitted and cross‐polarized light (XPL), reveal a clear structural and histological contrast between the thinner external layer of the ossicle and its thick basal plate.

**FIGURE 3:**
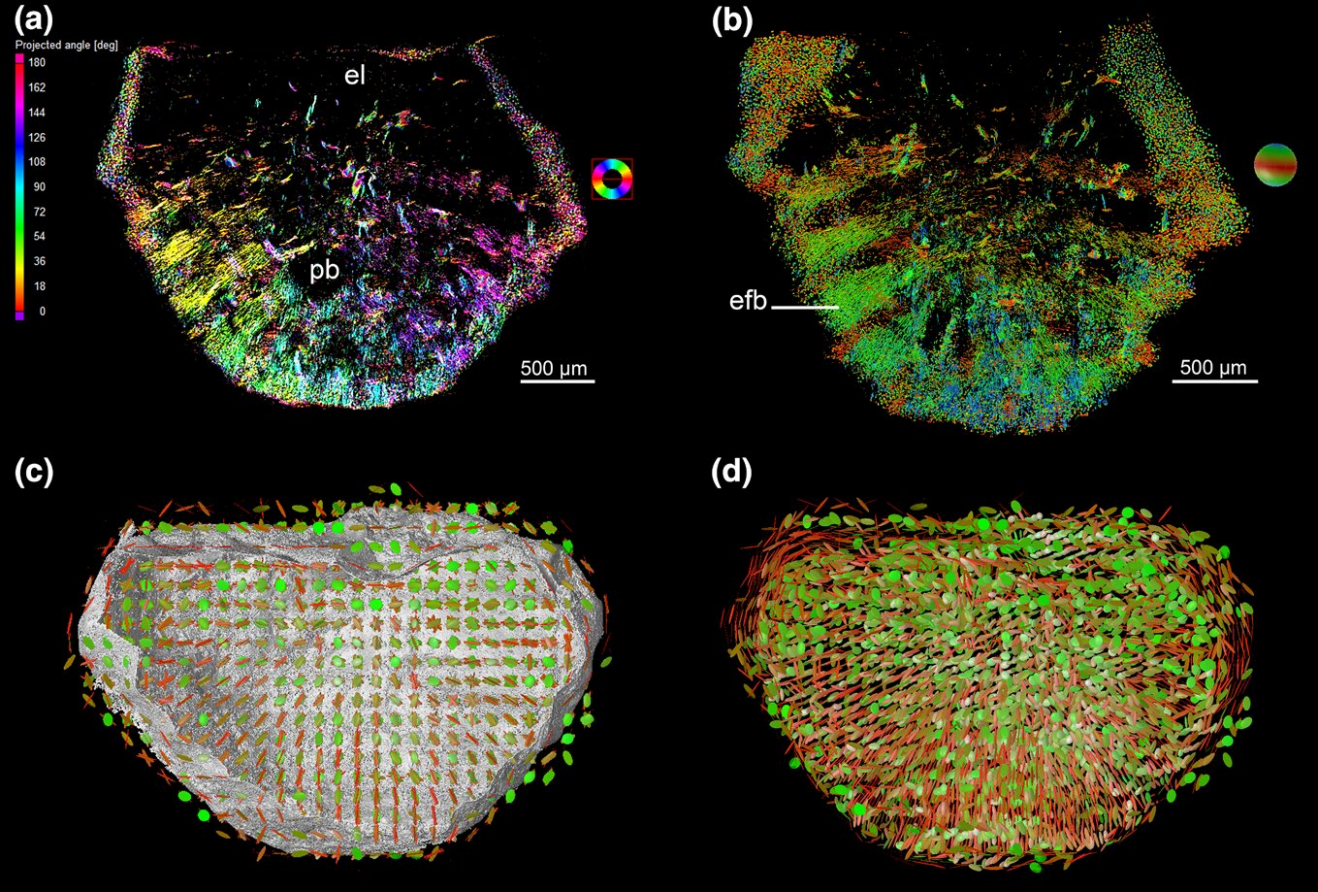
3D segmentation and orientation models of the fiber bundles in an ankylosaur ossicle (Museo de La Plata MLP 86‐X‐28‐1). (a) Virtual 3D longitudinal section, artificially colored with *plane projection planar orientation* (using *VG StudioMax*) to illustrate the radial orientation of some fiber bundles. They radiate from the upper region of the main vascular canals towards the surface of the basal plate (bp). The color coding follows regular intervals of projected angles. Note that the cell lacunae under the surface of the bone have been rendered to define the extant of the ossicle. (b) Virtual 3D longitudinal section, artificially colored with the option *space orientation deviation angle—rainbow—*using *VG StudioMax*, to highlight the enlarging pattern of the fiber bundles when reaching the surface of the ossicle. (c) 3D model of the ossicle with 150‐μm‐thick tensors representative of the orientation of the fiber bundles. Red tensors are orthogonal to the section plane while green tensors are parallel to the section plane. These tensors show two different patterns: The red tensors illustrate the radiating pattern of the radial fiber bundles and the green tensors the plywood pattern of the bone matrix. (d) Same 3D model as inC (but without the ossicle surface) with a slight oblique angle to highlight the radiating pattern. bp, basal plate; efb, enlarging fiber bundles; el, external layer.

The “external layer” appears as a smooth mineralized tissue whose surface elevations form the tubercles (Figure 2a_1_–d_1_). The “basal plate,” which forms the main part of the ossicle, is composed of interlaced bundles of closely packed mineralized fibers most probably made of collagen fibrils organized in a complex and ordered network. These interlaced collagen fiber bundles, the structural fiber bundles sensu Scheyer and Sander (2004), are organized in two orthogonal systems of fiber bundles that can be distinguished when ossicles are observed with XPL: (1) one horizontal system, and (2) another system approximately vertical (Figure 2a_2_–d_2_):

1. The horizontal system is itself composed of multiple layers of fiber bundles arranged into an orthogonal plywood‐like structure (Francillon‐Vieillot et al., 1990) (Figure 2a_2_,b_2_, Videos S1 and S2). In each layer, the fiber bundles are oriented in parallel but their direction varies from one layer to the next one with a rotating angle of about 90°, the horizontal layers are approximately parallel to the deep surface of the ossicle (Figure 2c_2_,d_2_), and actually form it.
2. The fiber bundles of the vertical system (Figure 3c) radiate vertically from the center of the ossicle at the level of the transition zone between the external layer and the basal plate and run towards the periphery of the basal plate. Their thickness increases from the center of the ossicle towards its periphery (Figure 2c_2_,d_2_, Figure 3a,b,d and Video S3).

#### 3.2.2 Cyclical deposition

The basal plate of ossicles shows distinct dark lines parallel to the deep surface, and to each other. These lines represent cyclical growth marks (CGMs) (Castanet et al., 1993). Some of those inconspicuous lines are located deep, in the central area of the basal plate (Figure 2c_1_,d_1_). CGMs seem more regularly distributed closer to the ossicle surface, where they form uninterrupted lines parallel to the basal surface of the ossicle. The distance between two adjacent CGMs seems larger in the center than in the periphery (black arrowheads in Figure 2c_1_,d_1_), thereby suggesting a decrease of radial growth with time.

#### 3.2.3 Vascularization

**FIGURE 4:**
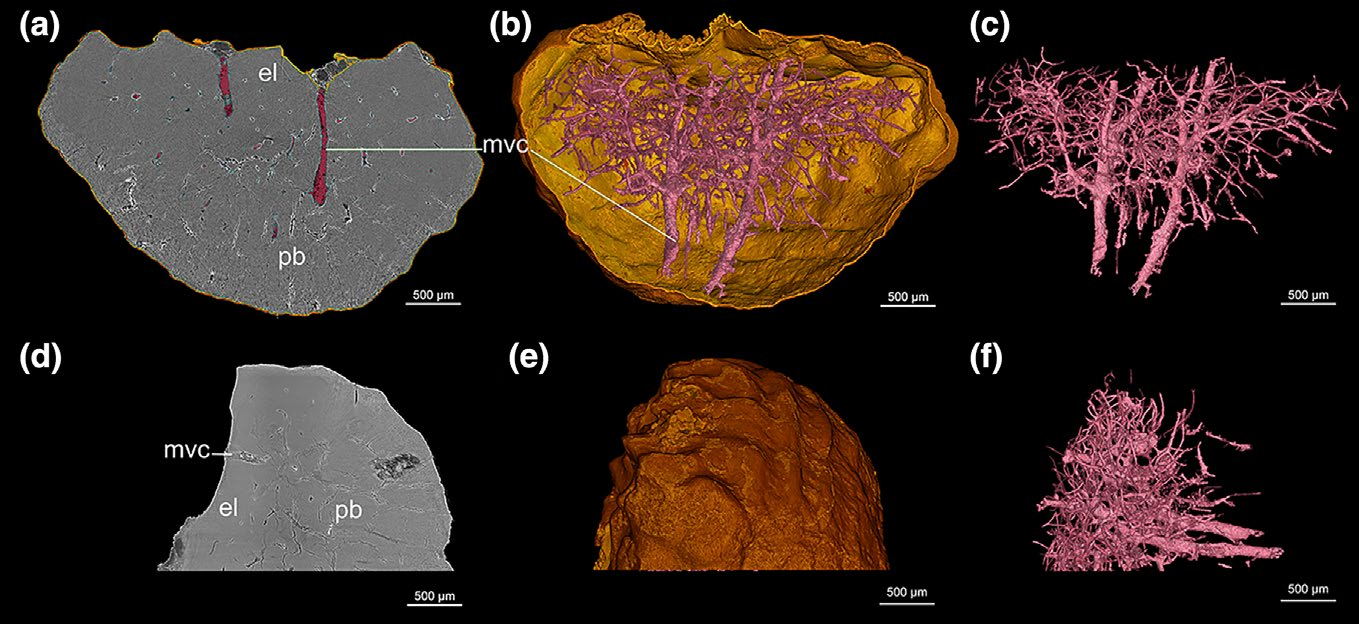
Propagation phase‐contrast synchrotron radiation micro‐computed tomography (PPC‐SRμCT) data showing the vascularization in an ankylosaurian ossicle (Museo de La Plata MLP 86‐X‐28‐1). (a) 50 μm thick virtual thin section revealing the vascular system departing from two larger central canals (mvc). (b) 3D model of the vascular mesh (in pink) surrounded by the surface of the ossicle (gold). (c) 3D segmentation of the vascular mesh illustrating its radial distribution around the two main central canals (in pink). The smaller canals remain restricted to the upper region of the basal plate while the two large canals cross the entire ossicle (from the external layer (el) to the tip of the basal plate (bp)). (d) 60‐μm thick cross‐section in the hemi‐scan of the osteoderm showing the distribution of the vascular mesh. (e) 3D model of the surface of the osteoderm. (f) 3D segmentation of the vascular mesh in the hemi‐osteoderm. bp, basal plate; el, external layer; mvc, main central vascular canals.

**FIGURE 5:**
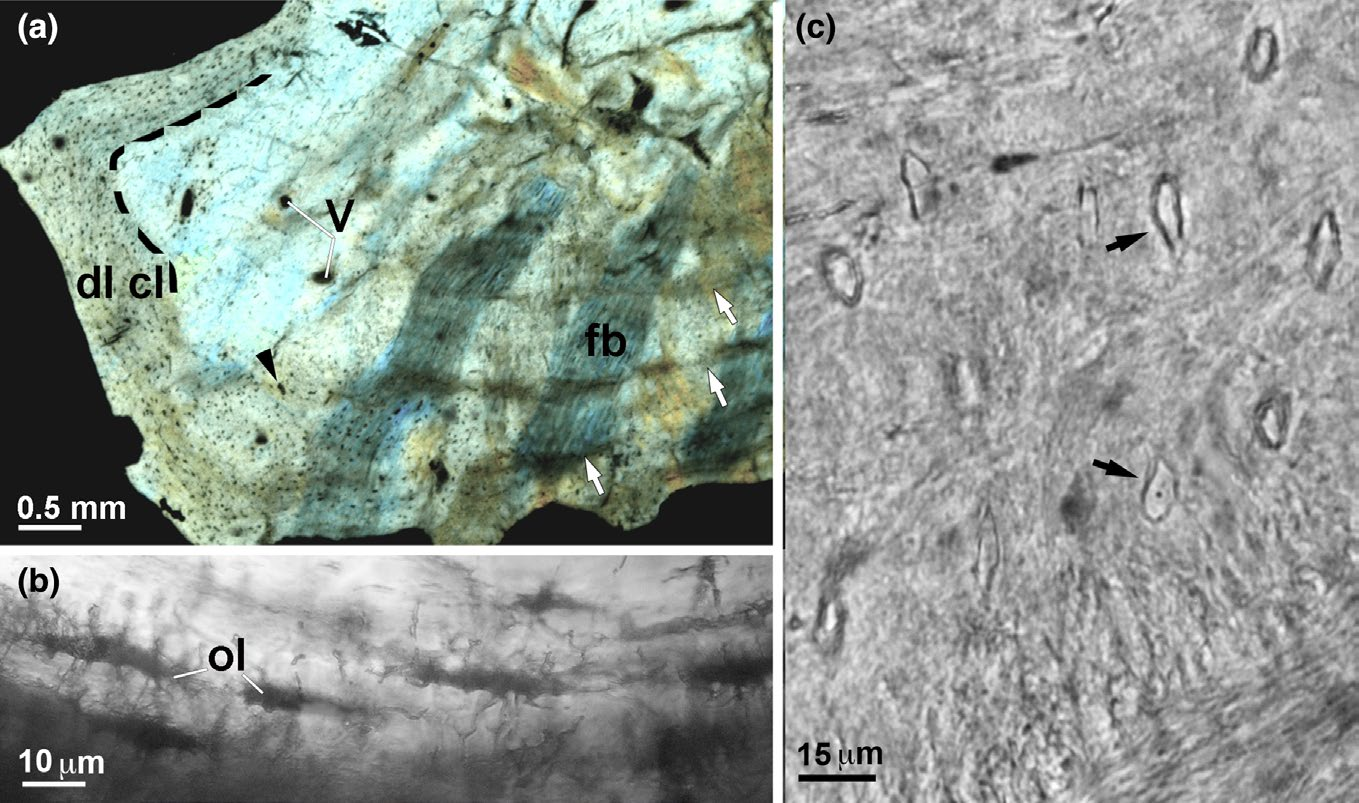
Cross‐section of the basal plate of an ossicle (Museo de La Plata MLP 86‐X‐28‐1). (a) Nomarski differential interference contrast microscopy. (b,c). Transmitted light (brightfield) microscopy. (a) Cell lacunae form a dense layer aligned along the surface of the basal plate (labeled as “dl cl”, for dense layer of cell lacunae). A primary vascular canal (arrowhead) is thinner than the vascular canal (V) lined by a thin layer of bone. Cyclical Growth Marks (CGMs, white arrows) are parallel to the surface of the basal plate. A vertical fiber bundle (fb) is perpendicular to the deep surface of the basal plate. (b) Spindle‐like lacunae of osteocytes (ol) with the characteristic canalicular organization observed in lamellar bone of secondary osteons. (c) Osteocytes lacunae with preserved content (black arrows) are interspersed among the fibers of the bundles in the basal plate.

3D images show that the central part of the basal plate contains two main vertical vascular canals that cross the entire ossicle from the external layer to the deepest region of the basal plate (Figure 4a–c). Each canal divides in turn into thinner branches approximately at the level of the upper middle part of the basal plate (Figure 4b–d,f). Arising from the main vertical vascular canals, a dense network of vascular canals occupies the central area of the basal plate (Figure 4b,c,f). The vascular canals become smaller as they approach the margin of the basal plate (Figure 4b,c,f). Some of them reach the margin of the ossicle and open outward (Figure 4b). The development of a dense vascular network in the central part of the ossicles observed on the 3D images is consistent with ossicle images from vertical sections showing that randomly distributed vascular canals are more numerous in the upper central part (Figure 4c,f). Apart from both large central canals, only few small vascular canals reach the deepest tip of the ossicle (Figure 4b). Some thin vascular canals cross the external layer to reach and open at the superficial surface of the ossicle (Figure 2a_1_,b_1_,d_1_; Figure 4a,b,d). The radiating vascular canals located in the central region of the basal plate appear as rounded or elongated elements in cross‐sections (Figure 2c_1_,c_2_,d_1_,d_2_, Figure 4b,c). Some of them form typical primary osteons (Figure 2c_1_) surrounded by concentric bone lamellae that do not interfere with the fiber bundles of the basal plate (Francillon‐Vieillot et al., 1990). Others that are not associated with concentric osteonal lamellae are simple primary vascular canals. Less numerous vascular canals have larger diameters and are involved in remodeling processes identified by the presence of a resorption line at the periphery of the osteonal bone tissue (Figure 2c_1_,c_2_,d_1_,d_2_). They are thus secondary osteons, where typical osteocyte lacunae are equipped with thin canaliculi containing the fine cytoplasmic processes of the living osteocytes (Figure 5b). They differ from the cells of the primary matrix of the basal plate that lack such canaliculi (Figure 5c).

#### 3.2.4 Fine organization of the external layer

**FIGURE 6:**
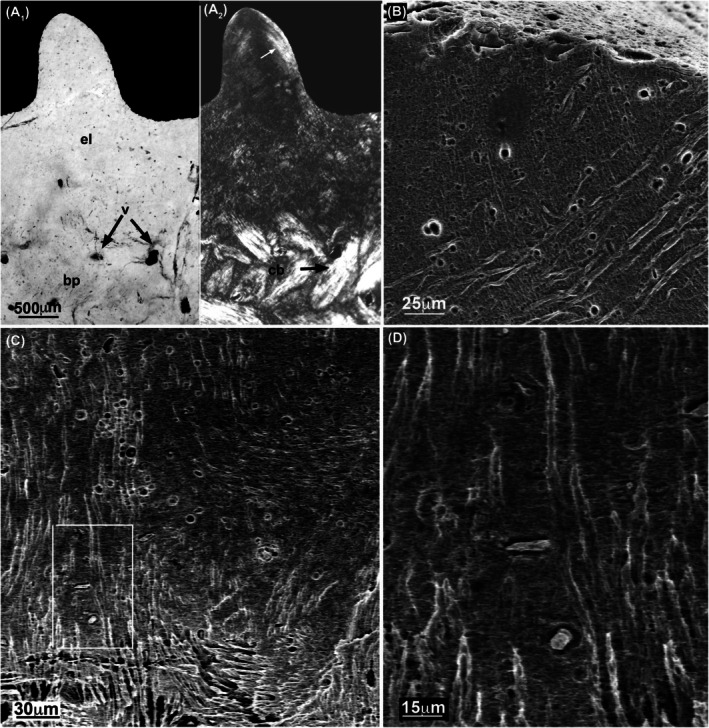
(a) Vertical section of an ossicle (Museo de La Plata MLP 86‐X‐28‐1). (a_1_) Transmitted light (brightfield) microscopy. Vascular canals distributed in the basal plate in the vicinity the external layer. (a_2_) Cross‐polarized light. The upper part of the basal plate is composed of interwoven structural fiber bundles. Thin fibrillar fascicules (white arrows) are observed at the tip of the elevations of the external layer. (b) Scanning electron microscopy (SEM). The superficial part of the external layer crossed by thin fibrils reaching the superficial surface of the ossicle. The amorphous material of the external layer contains randomly distributed holes. (c) SEM. Part of the external layer at the junction with the basal plate. Fibrils originating from the meshwork of the basal plate cross the external layer to reach the outer surface of the ossicle. (d) Insert of figure C showing cell lacunae containing cell remains. bp, basal plate; cb, structural fiber bundles; el, external layer; v, vascular canals.

The external layer is made of a material that appears homogeneous in vertical sections when observed with transmitted light microscopy (Figure 6a_1_). Only thin, inconspicuous lines are observed with XPL in some elevations forming the tubercles (Figure 6a_2_); they may correspond to loose bundles of mineralized fibers observed on SEM micrographs (Figure 6b–d). These thin mineralized fibers (about 3 μm in diameter) arise from the compact basal part of the ossicle and cross the homogeneous material of the external layer and reach the outer surface of the external layer (Figure 6b–d).

SEM micrographs show numerous holes randomly scattered through the external layer (Figure 6b,c). In cross‐sections, these holes appear circular of about 5 μm in diameter and when longitudinally sectioned they form elongated spaces (Figure 6b,c), sometimes arranged in succession to form short tunnels. It cannot be ruled out that these structures, restricted to the homogeneous tissue forming the external layer, are linked to post‐mortem bacterial or fungal actions. These holes differ from the less frequent ovoid lacunae randomly distributed within the whole external layer. Such lacunae have most probably housed cells whose remains are occasionally preserved (Figure 6b,d). The vitreous tissue that contains cells and crossed by bundles of mineralized fibers most probably collagen fibers, does not look like ordinary bone, neither like any ordinary hard tissue (such as dentine or enamel). It looks like the hypermineralized tissue called osteodermine observed in the osteoderms of some glyptosaurine squamates (de Buffrénil et al., 2010, 2011).

#### 3.2.5 Fine organization of the basal plate

**FIGURE 7:**
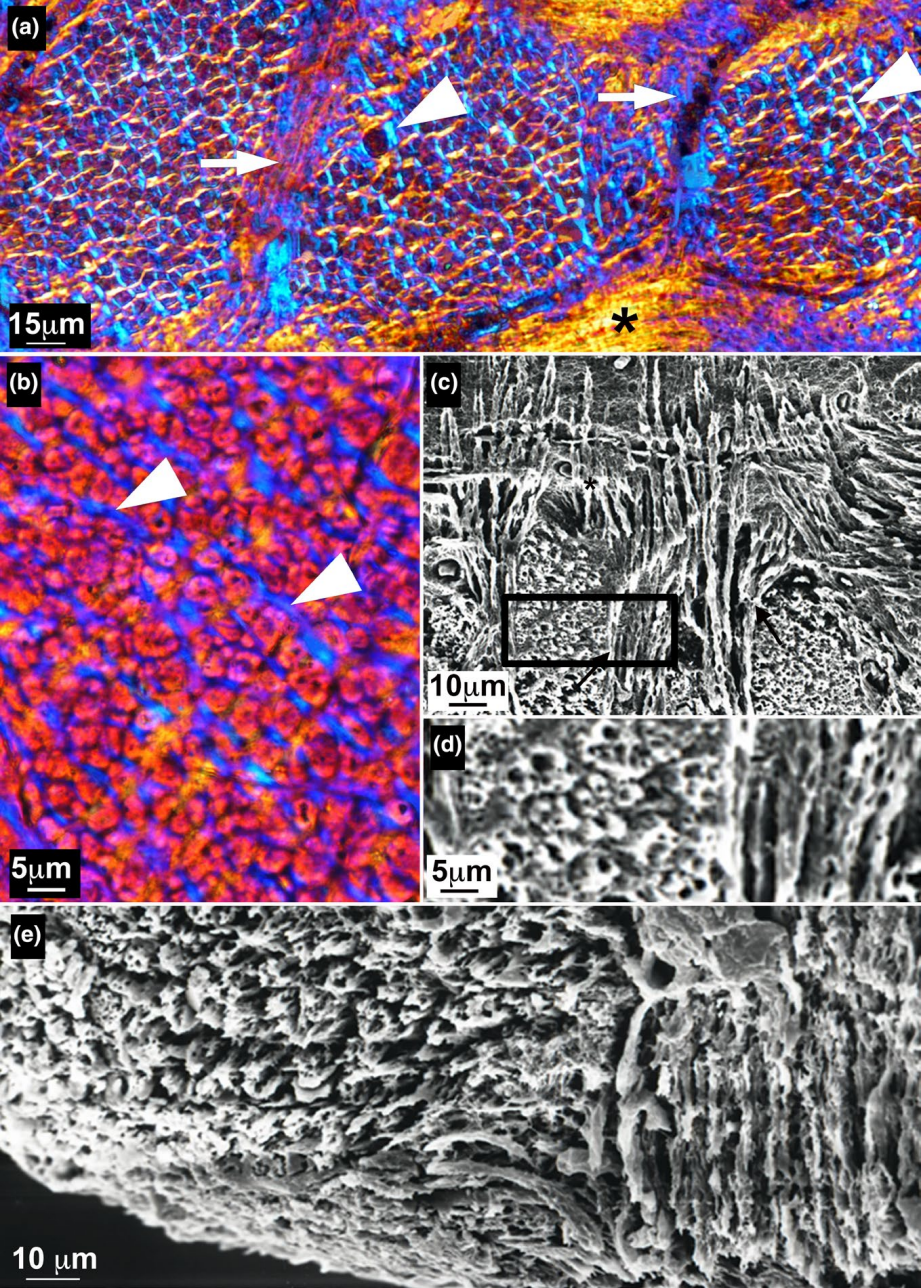
Basal plate (Museo de La Plata MLP 86‐X‐28‐1). (a) Cross‐polarized light (XLP). Cross‐section of structural fiber bundles. Thin fibers (arrowheads) are inserted among the cross‐sectioned fibrils of the bundles surrounded by thin bundles longitudinally sectioned (arrows). At the bottom, a longitudinally sectioned structural fiber bundle (asterisk). (b) XLP. Detail of a cross‐sectioned bundle showing cross‐sectioned fibrils surrounded by thin bundles (arrowheads). (c) Scanning electron microscopy (SEM). Two sets of fiber bundles are perpendicularly oriented forming an orthogonal meshwork surrounding a set of cross‐sectioned fiber bundles. (d) Tubular aspects of the fibrils. (e) Close‐up of the basal part of the ossicle.

Cross‐sections of the thick bundles of the horizontal system show an intricate network formed by sets of fiber bundles. The thick fiber bundles of the horizontal system are sheathed by thin sheets of fibers issued from the thick fiber bundles of the vertical system (Figure 7a,c). From the thin sheets emanate very thin fibrillar fascicules that interdigitate within the thick bundles of the horizontal system and are interspersed with the fibers of the bundles (Figure 7a,b, Videos S1 and S2). Light microscopy and SEM observations show that fibers of all the three systems of the complicated plywood‐like structure share the same pattern, they look like hollow, tubular, void fibers measuring about 3 μm in diameter (Figure 7b–e).

In the deep part of the basal plate, thick fiber bundles extend externally towards the margin of the ossicles (Figure 3a,b, Figure 7e, Video S3). The fibers extend perpendicular to the external surface of the ossicle (Figure 3, Figure 7e).

Numerous thinner (i.e., less dense) vertical bundles appear to interdigitate forming threads that interweave with the thicker bundles of the horizontal system (Figure 2a_2_,b_2_, Figure 3c,d, and Video [Link], [Link], [Link]). The thin threads penetrate within the horizontal bundles where they are inserted among the fibrils that appear tubular when cross‐sectioned (Figure 7a–c).

#### 3.2.6 Osteocyte lacunae

**FIGURE 8:**
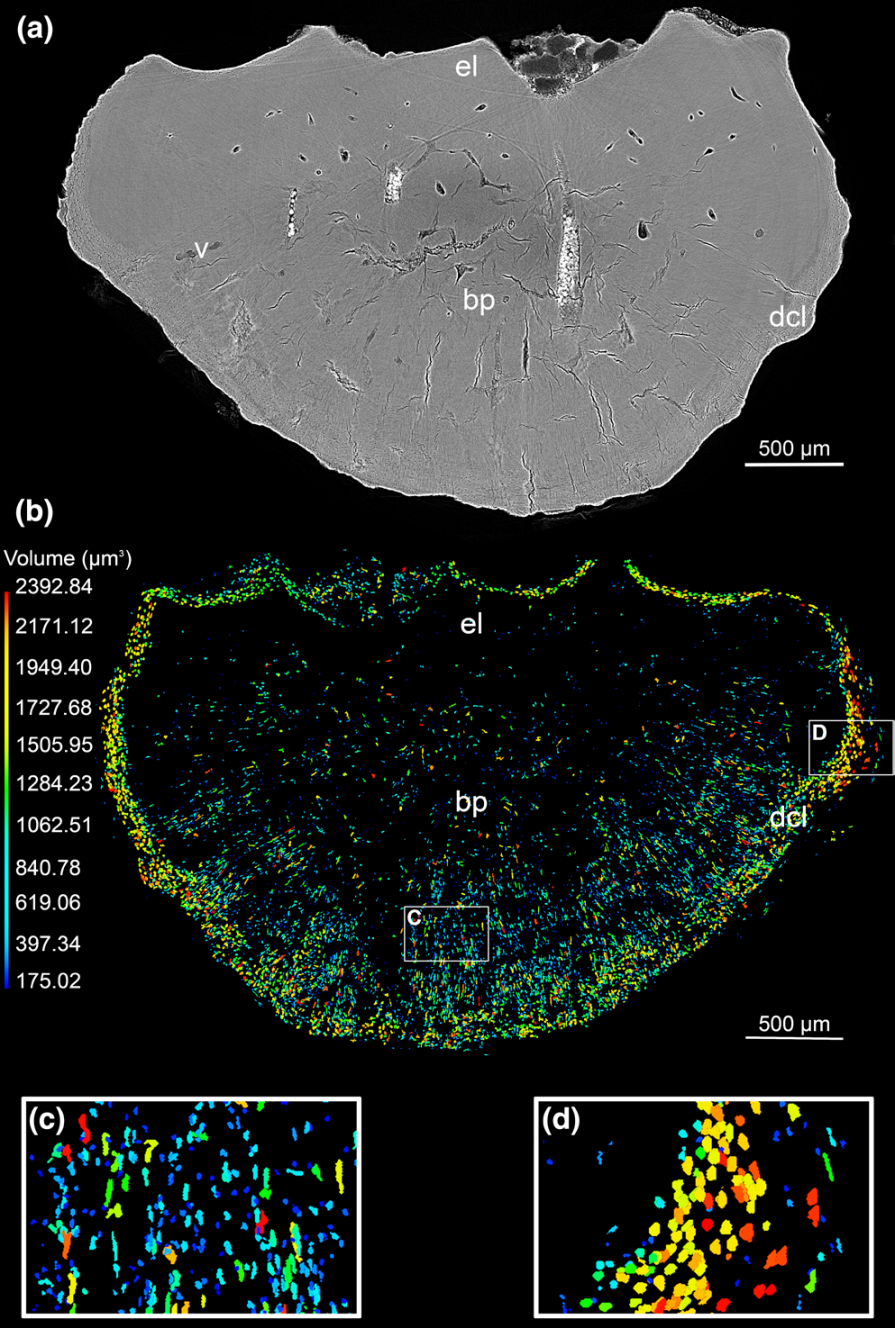
Propagation phase‐contrast synchrotron radiation micro‐computed tomography (PPC‐SRμCT) data showing the cell distribution in an ankylosaur ossicle (Museo de La Plata MLP 86‐X‐28‐1). (a) 50 μm thick virtual thin section revealing the peripheral distribution of large cell lacunae under the surface of the ossicle—dcl. (b) 3D virtual thick section of the cellular distribution in the ossicle and 3D volume measurement of these cell lacunae using *VG StudioMax* color coding. The cells are smaller in the core of the ossicle (blue‐green) than in periphery (green‐red). The cell spaces in the inner core of the basal plate (bp)—follow the pattern of the fiber bundles. (c) Zoom‐in on the basal‐plate cell spaces. (d) Zoom‐in on the cell lacunae under the ossicle surface.

Osteocyte lacunae are present in all regions within the ossicle, but especially below its surface: the most external region of the basal plate contains a dense layer of lacunae (labeled as dl cl on Figure 5a and dcl on Figure 8). They have housed cells whose remains are occasionally preserved (Figure 5b, Figure 6a,d). The osteocyte lacunae distribution in the ossicle is arranged in two main regions: (1) a dense layer of large osteocyte lacunae (which is 200 μm thick in the basal plate and 100 μm thick in the external layer) follows the surface of the entire ossicle (green, yellow and red‐cell spaces in Figure 8b,d); (2) the inner cores of the external layer and the basal plate however exhibit a more scattered organization of smaller cell lacunae (mainly blue‐cell spaces in Figure 8b,c).

1. In the dense layer (region framed in D, Figure 8), the cell lacunae are elongated and align along the entire surface of the ossicle (including both the deep and superficial surfaces) (Figure 8d). They have a volume ranging between 1000 and 2400 μm^3^ (Figure 8b).
2. In the inner core of the ossicle (region framed in C, Figure 8), the cell lacunae are only 175–1000 μm^3^ large (Figure 8b). They are slightly less numerous in the external layer than in the core of the basal plate (Figure 8b). They tend to follow the radiating bundles of fibers in the basal plate (Figure 8b).

## 4 DISCUSSION

Although the fossil material does not permit a direct observation of the mineralization processes, the resulting structure of the fossil material, in the framework of a comparison with extant groups (Sensale et al., 2014), sheds light on the processes occurring during osteoderm formation. In the ankylosaurian dinosaur *A. oliveroi*, the ossicles are composed of two layers: a thick basal plate made of a dense plywood‐like mineralized tissue and a thinner external layer of uncertain histological affinity. Recent histological description of ossicles from the same specimen as the one we observed (Cerda et al., 2019, Figure 2h–k) generally concur with de Ricqlès et al. (2001) and this also holds for the new observations of ossicle histology of undetermined ankylosaurs discovered in Southern Argentina (Paulina‐Carabajal et al., 2021, Figures 3 and 4).

A similar arrangement was described in the osteoderms of extant squamates, the Gekkonidae (Levrat‐Calviac & Zylberberg, 1986; Vickaryous et al., 2015), the Scincidae (Moss, 1969; L.Z. personal observations), and the Anguidae (Moss, 1969; Zylberberg & Castanet, 1985). The basal plate, that is, the main part of the ossicle, shows a dense plywood‐like tissue suggesting a greater contribution of the dermis in the first stages of mineralization than in crocodylian osteoderms that largely develop in the loose superficial dermis (Vickaryous & Hall, 2008).

### 4.1 Comparative structural considerations

#### 4.1.1 The external layer

The vitreous tissue of the external layer of the ankylosaurian ossicles shares some characteristics with the osteodermine described by de Buffrénil et al. (2010, 2011) in the osteoderms of extinct glyptosaurine anguids, and considered so far as unique to squamates (de Buffrénil et al., 2010, 2011; Vickaryous et al., 2015). Even if the terminology of this tissue remains debated, the term “osteodermine *sensu lato*” may be used here to name the superficial transparent vitreous tissue of the ossicles, pending further analyses of its composition, structure, and relationships.

This vitreous tissue shows a mono refringent reaction with XPL except at the tip of the tubercles in the ankylosaurian ossicles where thin bundles of collagen fibrils arise from the basal plate (Figure 6b). Such thin bundles of collagen fibrils crossing the external layer were also observed in the osteoderms of the extant geckos *Tarentola mauritanica*, *Tarentola annularis* and *Tarentola neglecta* where they were considered as Sharpey's fibers (Francillon‐Vieillot et al., 1990) connecting the osteoderms to the overlying dermal layers (Levrat‐Calviac, 1986–1987; Levrat‐Calviac & Zylberberg, 1986; Vickaryous et al., 2015). Apart from those Sharpey's fibers‐like collagenic extensions, the material composing the external layer of the ankylosaurian dinosaur ossicles presents an amorphous aspect on SEM micrographs (Figure 6). The vitreous layer of the ankylosaurian dinosaur ossicles differs from the osteodermine capping the osteoderms of fossil glyptosaurine squamates (de Buffrénil et al., 2010, 2011) in some aspects: the external layer of the ankylosaurian dinosaur contains cellular lacunae devoid of canaliculi and is crossed by rare thin capillary blood vessels (Figures 5 and 8). However, like the osteodermine of fossil glyptosaurines, the mineralized vitreous layer of ankylosaur ossicles is apparently lacking a fibrillar collagenous matrix. Hence, it cannot be considered as an osseous tissue.

Because of their topological and structural similarities, comparisons have been attempted between the squamate “osteodermine(s)” and various hypermineralized tissues covering the integumentary skeleton of various vertebrates: (1) amphibians (Gymnophiona: Zylberberg et al., 1980; Zylberberg & Wake, 1990); (2) non‐tetrapod vertebrates (resulting either from purely dermal (mesenchyme) origin, from a dermal‐epidermal production, or from an epidermal production only—Zylberberg et al., 1992; Donoghue, 2002; Donoghue et al., 2006; Sire et al., 2009; Vickaryous & Sire, 2009). Thus, hypotheses about the origin of squamate “osteodermine(s)” hint at the possibility that the overlying epidermal cells might have retained the ability to participate in the formation of such structures (de Buffrénil et al., 2011; Vickaryous & Sire, 2009). However, observations of the osteoderms of *T. mauritanica*, deeply inserted within the skin and without direct contact with the epidermis, but associated with dermal cells, led to favor a mesenchymal origin for gekkotan “osteodermine” (Levrat‐Calviac & Zylberberg, 1986; Vickaryous et al., 2015). Likewise, a mesenchymal origin might be hypothesized for the vitreous layer of the ankylosaurian dinosaur ossicle, despite the fact that relationships with the superficial soft tissues of the skin cannot be investigated in the fossil material.

#### 4.1.2 The basal plate

In the ankylosaurian dinosaur, the basal plate, the main part of the ossicle, is characterized by a highly organized system made of two sets of structural fiber bundles sensu Scheyer and Sander (2004). A similar highly organized architecture was observed in the “compacta” of nodosaurid osteoderms (Scheyer & Sander, 2004) such as *Patagopelta cristata* (Riguetti et al., 2022). The structural organization of the ossicles would therefore support the affinity of *A. oliveroi* with Nodosauridae (based on the diagnostic patterns published by Scheyer & Sander, 2004), thereby conforming to the phylogenetic hypothesis suggested by Salgado and Gasparini (2006) and Cerda et al. (2019), rather than the recent tree published by Soto‐Acuña et al. (2021).

The basal plate of the ossicles of *A. oliveroi* is composed of orthogonal systems of fiber bundles. The horizontal system is composed of two orthogonal layers of fiber bundles crossed by vertical fiber bundles (Figure 3). The peculiarity of the vertical fiber bundles is that their size increases from the center of the ossicle toward its periphery (Figure 3b). In that, they differ from the ossicles of the titanosaur *Saltasaurus loricatus*, which shows a similar three‐dimensional organization, but the diameter of the fiber bundles does not vary from the ossicle center toward its periphery (Cerda & Powell, 2010).

In the shell of fossil and living soft‐shelled turtles, where the structural bundles are organized in plywood‐like structure crossed by vertical bundles, the aspect of cross‐sectioned fibrils evokes that of tubular fibers (Scheyer et al., 2007). Similarly, longitudinal or cross‐sectioned fibers of the ossicle plywood‐like‐structure, most probably made of collagen fibrils, appear as tubular fibers (Figure 7). In addition to the orthogonal pattern of structuring the matrix of the basal plate, radiating fiber bundles run towards the surface of the ossicle (Figure 3). These bundles enlarge towards the surface of the ossicle. More studies are in progress in order to investigate whether apatite crystallites are oriented according to the collagen fibril orientation and structure (Landis et al., 2021).

Cell lacunae devoid of canaliculi are inserted within the fiber bundles, most cells are localized at the periphery of the basal plate. This location might be related to the growth of the ossicles that progresses radially by periodic fiber bundle mineralization, as suggested by the presence of CGMs (Figure 2). CGMs parallel to the bone surface and to each other show cyclic bone deposition, already observed in the metaplastic tissues of the squamate osteoderms (Levrat‐Calviac, 1986; Vickaryous et al., 2015). The cellular pattern in the inner core of the basal plate follows the radiating fiber distribution (Figure 8b,c) thereby suggesting a radiating expansion of the ossicle in size.

#### 4.1.3 Vascularization

In contrast with diminutive osteoderms where vascular spaces are commonly absent (Scheyer & Sander, 2009), the ossicles of *A. oliveroi* show a well‐developed vascular network despite their small size (Figure 4). The vascular canals are mostly found within the central part of the basal plate where they are ringed by primary and/or secondary lamellar bone tissue. They are arranged along two main large vascular canals longitudinally oriented along the ventro‐dorsal axis of the ossicle.

One key function of the bone vascular canals is to supply the bone forming cells with nutrients during growth. Actually several interpretations of the function(s) of bone vascular canals were hypothesized according to the diverse structural characteristics of bone as a tissue (Amprino, 1947; de Buffrénil et al., 1986). Primary osteons around thin blood capillaries are produced by the osteogenic activity of local cells. It has been shown in long‐bone compacta that a faster or a lower growth rate is correlated with a dense or sparse vascularization respectively (Amprino, 1947; Klein & Sander, 2008). Since primary vascular canals are incorporated into the bone during its deposition and do not develop after its formation, the large vascular mesh developed in the central part of the ossicle suggests that the growth of the ossicle was faster in the early stages of the ontogenesis and slowed down with time. Similarly, only the larger vascular canals located deep in the middle of the ossicle, that is, in its ontogenetically older part, had enough time to experience erosion/redeposition processes. They could therefore develop into typical secondary osteons which however form only a small amount of the total ossicle mass in this presumably not fully grown specimen.

From a developmental point of view, it can be hypothesized that it is the local development of a vertical vascular loop within the dermis that initiates the onset of fiber mineralization around it. Then, the radiating pattern of the vascularization in the ossicle that follows the radiating pattern of bundle fibers and osteocyte lacunae, consolidates the hypothesis of a radiating expansion of the ossicle until late in the development. More 3D histological investigations on osteoderms should be performed in relative species to *A. oliveroi* to verify if this is a common pattern, implying a common ossicle developmental process.

### 4.2 Ossicle growth as a whole

In the preliminary histological description of the ossicles, de Ricqlès et al. (2001) questioned the straightforward interpretation of a simple metaplastic origin of the basal plate, via direct ossification of the preexisting dermis *stratum compactum*. While acknowledging the obvious “metaplastic‐like” tissue pattern forming the basal plate of the ossicle, this interpretation was questioned in view of apparently conflicting evidence. The main reason for this questioning was the apparent radial growth in diameter of the fibrous bundles of the 3D plywood‐like system (Figure 3a,b). Indeed, the radial growth and expansion of the ossicles by simple centrifugal mineralization of the preexisting fibers via metaplasia would be non‐problematic, if the fibers themselves kept their original dimensions and diameters during the ossicle growth process. In such case, the number of fibers would increase in length to accommodate the osteoderm radial size increase without changing the size of the fibers themselves. However, this appears not to be the case at least in the vertical system (Figure 3a,b).

During the ossicle growth, the unmineralized dermis progressively gets further away from the ossicle “center of radiation,” that is, the ontogenetically oldest part from where the ossicle originated. The general pattern of fibrillar organization of the ossicles indeed suggests a radially additive growth, also evidenced by the successive CGMs (Figure 2), although it has not been possible to substantiate quantitatively a significant increase in the diameter of the bundles of the meshwork in radial directions. This takes into account the difficulty to assess the spatial repartition of the bundle diameters, caused by the haphazard section orientations of many ossicles. Nevertheless, this likelihood of a radial increase in bundle diameters is supported by our observations (for instance the obvious radial divisions of the vertical bundles as they get closer of the ossicle periphery on Figure 3b) and is also noted and figured by more recent descriptions (Cerda et al., 2019, Figure 2h–k; Paulina‐Carabajal et al., 2021, Figure 4).

**FIGURE 9:**
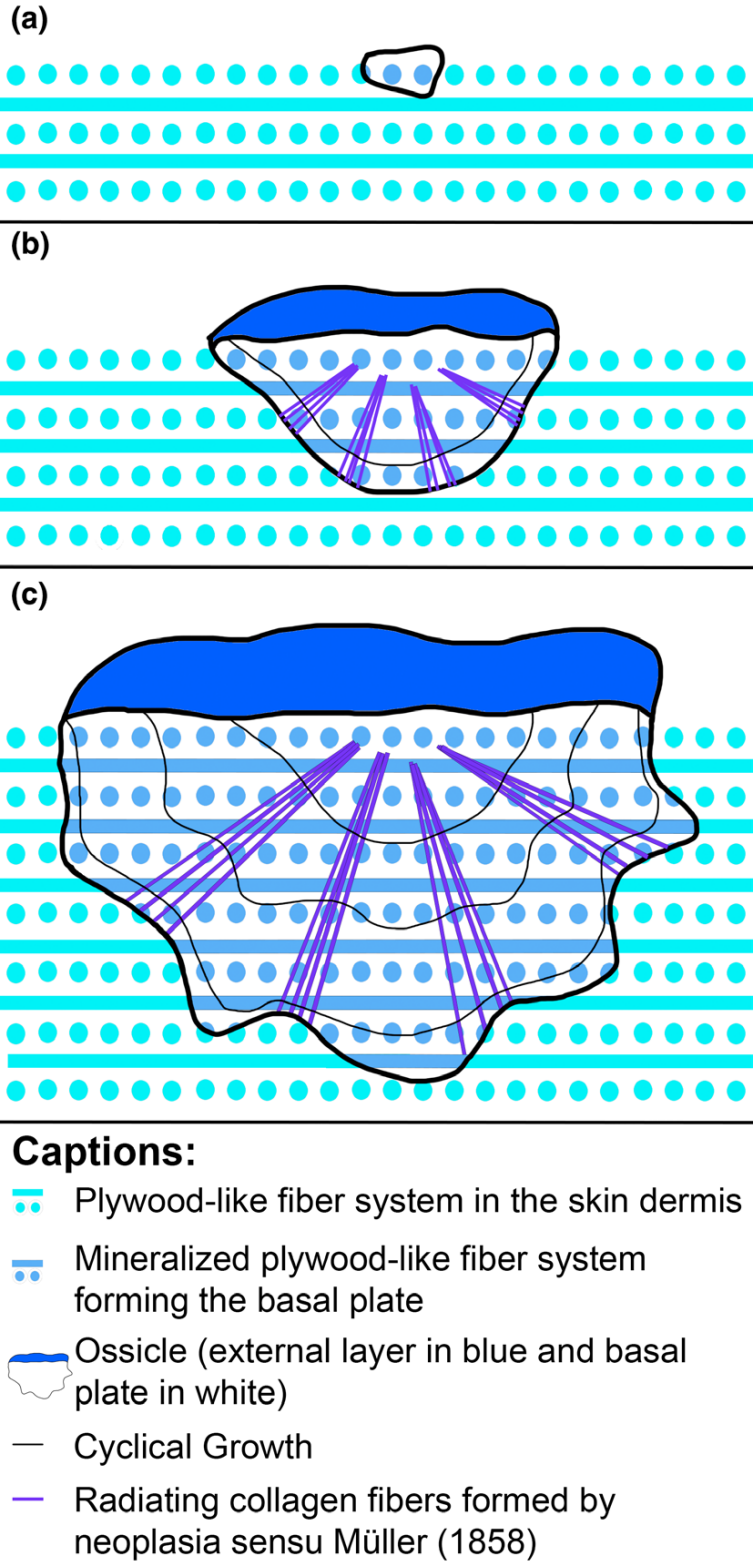
Schematic drawing summarizing the development of a dermal ossicle of *Antarctopelta oliveroi* with three steps illustrated (a–c). (a) Initiation of the ossicle development from the mineralization of the plywood‐like fiber system in the dermis (metaplasia sensu Müller, 1858). This forms the upper region of the basal plate. (b) Formation of the vitreous external layer through neoplasia sensu Müller (1858). Extension of the mineralization front through metaplasia sensu Müller (1858) by mineralizing successive layers of plywood‐like fibers of the dermis onto the preexisting surface of the basal plate. Extension of the basal plate through neoplasia sensu Müller (1858) by forming new radial fiber bundles along the ossicle growth process. (c) Cyclical deposition of the ossicle, thereby forming cyclical growth marks (CGMs).

Thus, we observe an apparent paradoxical situation where the preexisting dense fiber system of the dermis ossifies directly (metaplasia) but nevertheless simultaneously records a diameter expansion of its fiber bundles along their radial growth (Figures 3 and 9). Our discussion attempts to show that the occurrence of a diametral growth of the collagen fiber bundles is possible in these ossicles and even not as contradictory to the metaplastic interpretation as it may seem.

Indeed, if one takes into consideration the *time dimension* of the dermis ontogeny as the animal gets larger and larger, it may not be appropriate to consider the *stratum compactum* as a “frozen” preexisting and non‐changing structure. The unmineralized parts of the dense dermis can grow following various patterns of intussusceptive (from within) as well as additive (on a surface) growth, including cells multiplications, fiber bundles formation, expansions, additions, and divisions (Krejsa, 1979). In other words, the *stratum compactum* would not be a preexisting template fixed permanently before that mineralization takes place, but rather a living template experiencing itself addition and expansion (Figure 9).

In the case of a metaplastic ossification, the radiating expansion of mineralization from an initial locus would thus sequentially “freeze” the expansion of the *stratum compactum* itself, as the skin expands and thickens according to the whole organism growth. In other words, the radially additive mineralization of the ossicle would also record the intussusceptive growth and expansion of the non‐mineralized dermis. Hard‐tissue sections always actually record space/time relationships in growth processes.

How could this progressive expansion of the *stratum compactum* be realized? The hypothesis of different modes of formation of the ossicles, rather than by strict direct metaplasia, is substantiated by the observation of numerous large cells towards the periphery of the ossicle, forming a dense “crown‐like” halo close to its surface (Figure 8b,d). This suggests that the unmineralized *stratum compactum* immediately external to the mineralization front (= the surface of the ossicle) was densely permeated by cells associated to the differentiation of supplementary threads of collagen fibers and to the forthcoming mineral deposition. Many cells became entrapped within the mineralization deposit (hence the cell “crown” close to the basal ossicle surfaces, Figure 8b,d) and most would later degenerate and shrink in the more internal (older) parts of the ossicle (hence their lower density there, Figure 8b). The nature of the cells (fibroblasts or preosteoblast‐like or others) cannot be ascertained, but they do not mature into typical osteocytes, as evidenced by the lack of canaliculi (Figure 5c). Accordingly, the new fiber bundles differentiating in the unmineralized dense dermis close to the ossicle surface keep the typical « metaplastic‐like » pattern (Figure 9).

If the structure of the ossicle basal plate originates in part from metaplastic ossification and reflects the spatial arrangement (and expansion) of the unmineralized fibers forming the *stratum compactum* of the dermis, the formation of a vitreous layer at the surface of the ossicles, on the other hand, can hardly be considered as resulting from metaplasia.

As previous authors emphasized, not all osteoderms of tetrapods originated exclusively via metaplastic ossification (Main et al., 2005; Scheyer & Sander, 2004; Vickaryous et al., 2015; Vickaryous & Hall, 2008; Vickaryous & Sire, 2009). Although metaplastic ossification may certainly participate in osteoderm ossification in tetrapods, it is not necessarily the only mode of development. Other processes such as neoplasia sensu Müller (1858) may be involved as well (de Buffrénil & Zylberberg, 2021). For example, Reid (1996) hypothesized that metaplasia would initiate the development of the osteoderms (based on the observation of the conserved structure of the skin), and further ossification processes (such as neoplasia sensu Müller, 1858) could occur afterwards. Thus, metaplastic and osteoblastic (i.e., neoplastic sensu Müller, 1858) tissues could coexist within dinosaurian osteoderms with possibly different functions and different expressions along ontogeny (Cerda, Garcia, et al., 2015; Horner et al., 2016; Main et al., 2005). However, there is no evidence that a simultaneous occurrence of metaplasia (by mineralization of preexisting dermal fibers) and neoplasia (differentiation and mineralization of new fiber bundles, Müller, 1858) would change extensively during the growth of the ossicles.

### 4.3 Functional hypotheses

The ossicle is composed of two layers: (1) a superficial possibly hypermineralized layer; and (2) a fibrous basal plate with a combination of plywood and radiating collagen fibers. Each of these layers likely has specific and complementary functions. The peculiar, possibly hypermineralized, structure of the external layer would be harder and more resistant to fractures than the basal plate (Martin et al., 1998). However, the highly mineralized nature of it would also have a greater brittleness (Currey, 2004). This means that it is unlikely that a crack would form in this tissue but, if it did eventually form, it would probably spread quite rapidly (Martin et al., 1998). To avoid destroying the entire ossicle after a direct shock/bite, the presence of an underlining mineralized component with a different molecular organization, and density seems advantageous. The architectural organization of the structural fiber bundles, crossing each other in the thick ossicle basal plate at the manner of a plywood‐like material, could play this role. Thanks to their multidirectional organization, bone‐matrix plywood configurations are known to enhance bone's ability to resist shear stresses in torsion and compression loading (Martin et al., 1998). The plywood‐like basal plate of this ankylosaurian ossicle would therefore have complementary biomechanical advantages to the hypermineralized external layer against crack, shocks or bites.

In addition, fiber bundles of the basal plate might have likely continued into the surrounding dermis anchoring the ossicle within the dermis and linking it to the neighboring ossicles with which they would form a pavement with the larger osteoderms as in extant reptiles (Levrat‐Calviac & Zylberberg, 1986). The basal plate as a whole, may be considered entirely as a functional analogous structure to Sharpey's fibers, with an anchoring function in the neighboring unmineralized dermis (Benjamin et al., 2002; Scheyer & Sander, 2004). Contrary to the massive, compact osteoderms (formed through metaplasia only), the development of small ossicles—composed of two types of tissues of different densities (formed by metaplasia and neoplasia sensu Müller, 1858)—would not only make it possible to absorb small direct shocks but it would also give a real flexibility to the ossicle to resist stretching of the skin during movements of the animal's body.

## 5 CONCLUDING REMARKS

This study expands our knowledge regarding the 3D histology of the button‐like ossicles of ankylosaurs and provides new data on the spatial organization of the collagen fiber bundles, on cells localization and on the vascularization. Our data support that the ossicles were at least partially formed by metaplasia sensu Haines and Mohuiddin (1968) and suggest that this process is supplemented by the cell‐induced differentiation of new fiber bundles laid down immediately prior to their incorporation into the fibrous system and its mineralization. This process looks more akin to neoplasia (de novo formation of a tissular structure without a preexisting tissue template, Müller, 1858) than to metaplasia. Nevertheless the new fiber bundles keep a structure and organization similar to those of a typical metaplastic tissue, to which they become superficially added. This process explains the progressive diametric expansion of the fiber bundles as the whole ossicle expands centrifugally. It is likely that the system of horizontal fiber bundles in the ossicle is more involved in direct metaplasia while the vertical fiber system is more involved in the neoplastic process we suggest.

The probability that other mechanisms are required to account for the structure of the ossicles deserves further discussion. The superficial part of the ossicle is formed by a peculiar mineralized tissue that is not bone (“osteodermine” sensu lato), and it requires further studies to be properly understood. Ankylosaur osteoderms are distinctive among dinosaur genera and species. Since ankylosaur dinosaurs are widely believed to have been solitary animals, even isolated ossicles are important fossils to collect, demonstrating the local occurrence of ankylosaur taxa in specific stratigraphic contexts (Paulina‐Carabajal et al., 2021).

## AUTHOR CONTRIBUTIONS

L.Z. and A.de R. designed the study; L.Z., S.S., and P.T. designed the experiments; L.Z., S.S., and P.T. performed the experiments; S.S. and J.P. segmented the 3D histological data and produced the 3D figures; L.Z. produced 2D figures and wrote the first version of the manuscript with inputs from A.de R. and S.S.; all the authors edited the manuscript and agreed on the final version of the article.

## Supporting information


Video S1.

Video S2.

Video S3.

## Data Availability

The data that support the findings of this study are openly available in an online repository in the *ESRF heritage database for palaeontology, evolutionary biology and archaeology* (http://paleo.esrf.eu/)
